# The EPICC Family of Anti-Inflammatory Peptides: Next Generation Peptides, Additional Mechanisms of Action, and *In Vivo* and *Ex Vivo* Efficacy

**DOI:** 10.3389/fimmu.2022.752315

**Published:** 2022-02-09

**Authors:** Neel K. Krishna, Kenji M. Cunnion, Grace A. Parker

**Affiliations:** ^1^ Division of Research, ReAlta Life Sciences, Norfolk, VA, United States; ^2^ Department of Pediatrics, Children’s Hospital of The King’s Daughters, Norfolk, VA, United States; ^3^ Children’s Specialty Group, Norfolk, VA, United States; ^4^ Department of Pediatrics, Eastern Virginia Medical School, Norfolk, VA, United States

**Keywords:** EPICC, PIC1, RLS-0071, complement, myeloperoxidase, neutrophil extracellular traps, antioxidant, neutrophil

## Abstract

The EPICC peptides are a family of peptides that have been developed from the sequence of the capsid protein of human astrovirus type 1 and previously shown to inhibit the classical and lectin pathways of complement. The EPICC peptides have been further optimized to increase aqueous solubility and identify additional mechanisms of action. Our laboratory has developed the lead EPICC molecule, PA-dPEG24 (also known as RLS-0071), which is composed of a 15 amino acid peptide with a C-terminal monodisperse 24-mer PEGylated moiety. RLS-0071 has been demonstrated to possess other mechanisms of action in addition to complement blockade that include the inhibition of neutrophil-driven myeloperoxidase (MPO) activity, inhibition of neutrophil extracellular trap (NET) formation as well as intrinsic antioxidant activity mediated by vicinal cysteine residues contained within the peptide sequence. RLS-0071 has been tested in various *ex vivo* and *in vivo* systems and has shown promise for the treatment of both immune-mediated hematological diseases where alterations in the classical complement pathway plays an important pathogenic role as well as in models of tissue-based diseases such as acute lung injury and hypoxic ischemic encephalopathy driven by both complement and neutrophil-mediated pathways (*i.e.*, MPO activity and NET formation). Next generation EPICC peptides containing a sarcosine residue substitution in various positions within the peptide sequence possess aqueous solubility in the absence of PEGylation and demonstrate enhanced complement and neutrophil inhibitory activity compared to RLS-0071. This review details the development of the EPICC peptides, elucidation of their dual-acting complement and neutrophil inhibitory activities and efficacy in *ex vivo* systems using human clinical specimens and *in vivo* efficacy in animal disease models.

## Introduction

The innate immune system is the body’s first line of defense against microorganisms. It consists of both humoral and cellular mechanisms that are triggered immediately upon infection. Two of the primary components of innate immunity are phagocytic cells (neutrophils and macrophages) and blood proteins (the complement system). While complement and neutrophils are an essential host defense against invasive microbes, dysregulation of the innate immune response plays a prominent role in a variety of inflammatory and autoimmune diseases. In this review article, we describe a novel class of anti-inflammatory peptides (termed “EPICC”) that possess a unique dual-acting mechanism of action: inhibition of both complement and neutrophil-mediated activation. The discovery, *in vitro* and *ex vivo* characterization as well as the *in vivo* activity of these peptides in pre-clinical blood and tissue-based disease models, will be discussed along with their implications for therapeutic use in inflammatory disease processes.

## Discovery of the EPICC Peptides

### Human Astrovirus

Human astrovirus serotype 1 (HAstV-1) is a non-enveloped, icosahedral virus with a single-stranded, 7 kilobase positive-sense RNA genome that is an endemic global pathogen (1). HAstV-1 is the prototypic family member and the most well studied of the *Astroviridae* (2). Human astroviruses infect young children and unlike other enteric pathogens, cause a non-inflammatory, self-limiting gastroenteritis (3). The lack of inflammation associated with astrovirus infection led us to hypothesize that the virus capsid may directly interact with components of the host immune system resulting in a blunted inflammatory response as reported in humans and other animal infections. The HAstV-1 capsid is composed of 180 copies of a single capsid protein of 787 amino acid residues and structural predictions using three-dimensional position-specific scoring matrix (3D-PSSM) software (4) revealed that the capsid protein possessed weak homology to human complement regulatory proteins. This led us to examine if the HAstV-1 capsid protein could directly modulate the human complement system.

### Suppression of the Complement System by HAstV-1 Capsid Protein

**Figure 1:**
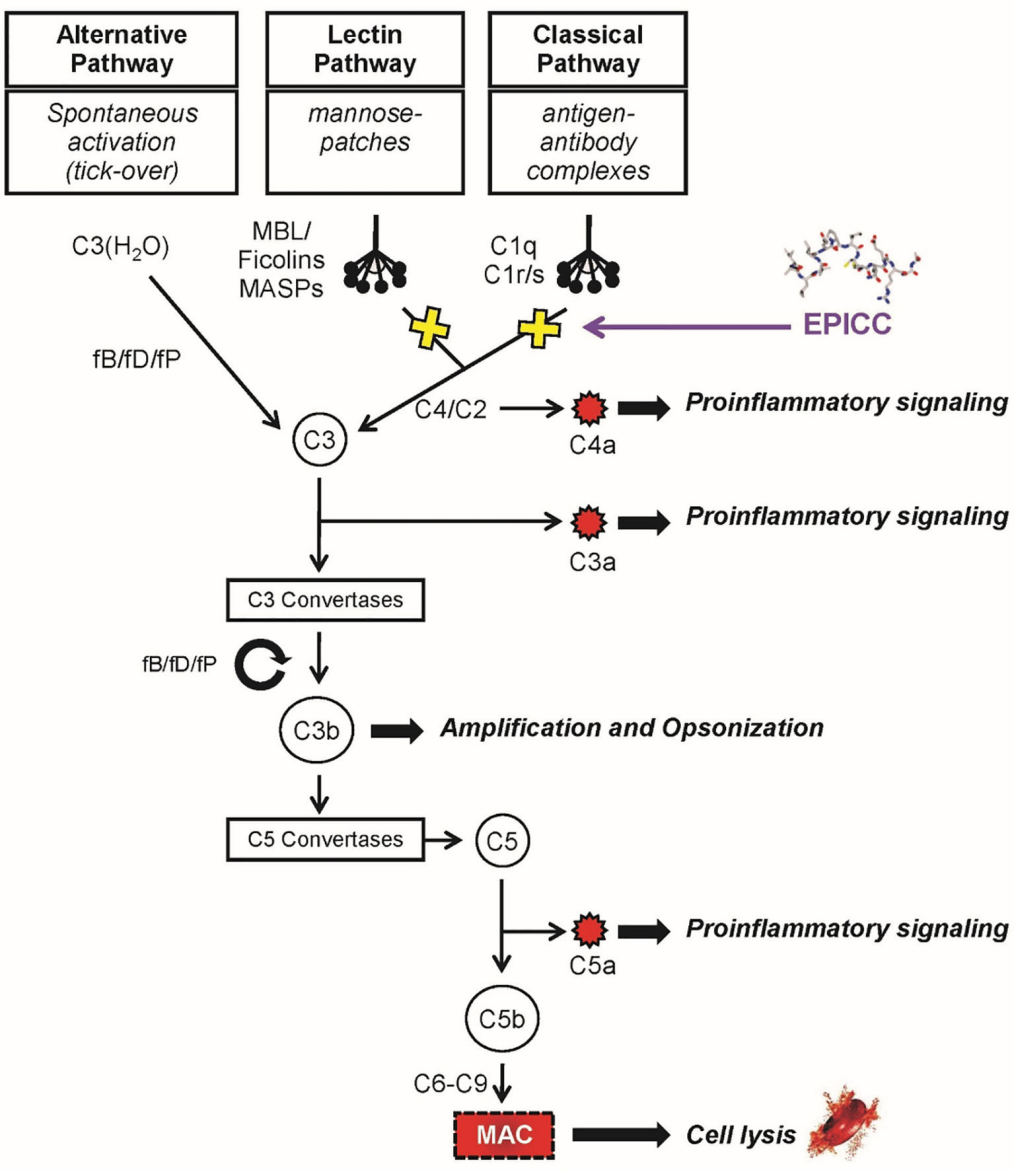
The complement system. The alternative, lectin and classical pathways with the main protein factors and effector functions are indicated. The EPICC peptides specifically bind to the initiator molecules of the classical and lectin pathways, C1q and MBL, respectively, inhibiting complement activation and downstream effector functions. The EPICC compounds do not interfere with the essential immune surveillance functions provided by the alternative pathway.

The complement system constitutes part of the humoral innate immune system and is comprised of over 30 plasma and cell bound proteins (5). The complement system can be activated through three pathways: classical, lectin, and alternative (6) (**Figure 1**). These pathways are enzymatic cascades that converge at C3 and are normally tightly controlled by soluble and cell bound regulatory proteins (5). The main activities of the complement system are to provide a first line of defense against infection, bridge the innate and adaptive immune systems, and maintain immune homeostasis by clearing apoptotic cell debris (7). The classical complement pathway is activated *via* antibody bound to the surface of a microbe or immune complexes (8) as well as in an antibody-independent manner, by molecules such as C-reactive protein or pentraxins that directly activate the classical pathway (9–14). Similar to the classical pathway, the lectin pathway is composed of soluble pattern-recognition molecules, that activates upon recognition of mannose groups on the surface of microbes (15). The alternative pathway is activated when C3 directly recognizes microbial surface structures (16) and amplifies the activation of both the classical and lectin pathways (5). Activation of any of these three pathways results in generation of the anaphylatoxins C3a and C5a as well as the membrane attack complex (MAC) which results in cellular lysis. This cascade of complement activation results in microbial destruction through multiple mechanisms, including the lysis of infected cells and certain pathogens, opsonization and phagocytosis, removal *via* immune complexes, enhanced priming of T and B cells, and mediation of a robust inflammatory response *via* leukocyte chemotaxis to the site of infection (7, 11). However, abnormal activation of any of these pathways can lead to significant tissue damage and is associated with inflammatory and autoimmune diseases (17–20).

Classical complement activation is mediated *via* the multimolecular complex, C1 (8). C1 is comprised of the charge pattern recognition protein, C1q, and serine protease tetramer, C1s-C1r-C1r-C1s. C1q is composed of 6 copies of three protein chains (A, B and C) that together form an 18-chain molecule with a collagen-like region (CLR) and a globular head region (GHR). The CLR has a semi-flexible “hinge” which houses the C1s-C1r-C1r-C1s tetramer (21). Binding of IgM or clustered IgG to the GHR induces a conformational change in C1q resulting in activation of the C1s-C1r-C1r-C1s tetramer and sequential activation of C4 and C2 of the classical pathway and downstream effector functions of the complement system. Initial studies in our laboratory demonstrated HAstV-1 capsid protein was able to potently inhibit complement activation in a standard hemolytic assay. In this assay, human serum is added to antibody sensitized red blood cells (RBC) which activates the classical pathway resulting in MAC formation on the RBCs and hemolysis. HAstV-1 capsid protein inhibited hemolysis to a similar degree as cobra venom factor (CVF), the gold standard complement inhibitor (22). We further demonstrated that HAstV-1 capsid protein binds to the hinge region of the CLR of C1q, interrupting the interaction of the cognate serine protease complex (C1s-C1r-C1r-C1s) and thus inhibiting classical complement pathway activation (23). The astrovirus capsid protein also was found to bind the initiator molecule of the lectin pathway, mannose binding lectin (MBL) protein which shares structural homology to C1q and inhibit activation of the lectin pathway of complement (23). HAstV-1 capsid protein had no inhibitory activity on the alternative pathway which provides essential immune-surveillance functions against invading pathogens (22, 24).

### Identification of the EPICC Peptides

The capsids of icosahedral RNA viruses perform multiple functions in the virus life cycle such as self-assembly, viral RNA packaging, host receptor binding, viral particle disassembly and immune evasion. We thus hypothesized that the region of the HAstV-1 capsid that possesses complement inhibitory activity would map to a limited amino acid sequence within the 787 amino acid capsid protein. Based on sequence alignment data, we identified a 60 amino acid region of the HAstV-1 capsid protein that had limited homology to human neutrophil peptide-1 (HNP-1) (25) which had previously been identified as an inhibitor of the classical and lectin complement pathways (26). This region of the HAstV-1 capsid was subsequently demonstrated to be exposed on the surface of the mature astrovirus particle by X-ray crystallography (27) and thus available to interact with host immune factors. It was also demonstrated that human astrovirus blunts the intracellular C3 autonomous immune response as assessed by reduced NF-kB levels which the authors attributed to HAstV inhibition of C1 (28).

The active region of this peptide was further defined to a peptide of 15 residues within the HAstV-1 capsid protein and the sequence rearranged to improve the amphipathic nature of the molecule by reorganizing the hydrophobic residues to the N terminus and the hydrophilic residues to the C terminus, creating the Polar Assortant (PA) peptide (amino-acid sequence IALILEPICCQERAA) (29). The PA peptide was shown to have superior complement inhibiting activity in the hemolytic assay compared to previous astrovirus-derived peptide derivatives and is unique in nature with no known homologies to other natural proteins or peptides (29–31). Increased functionality of PA was not improved by the systematic substitution of positively or negatively charged residues using alanine, arginine, or glutamic acid scans or deletion of a single residue at the N- or C-termini which resulted in decreased ability of PA to inhibit the classical complement pathway (29, 31). The PA molecule is the base peptide of the EPICC family of peptides, named for the conserved central five amino acids (IALILEPICCQERAA).

PA was subsequently shown to inhibit ABO mediated RBC incompatibility, a classical complement pathway driven disease process, which was demonstrated in an *ex vivo* hemolytic assay using incompatible human type O serum and human AB RBCs, *ex vivo* using serum isolated from the blood of rats, and was also shown to inhibit complement activity when administered intravenously into rats (29). The cross-species activity of PA and other EPICC derivatives (31) have been essential for the pre-clinical development of the EPICC derivatives as it allows the peptides to be tested in animal models of complement and neutrophil-mediated disease. PA is not water soluble and requires solubilization in dimethylsulfoxide (DMSO). To increase solubility in aqueous solution for *in vivo* administration, PA was synthesized to include a monodisperse, 24-mer polyethylene glycol (PEG) unit linker on either the N terminus (dPEG24-PA), C terminus (PA-dPEG24), or N and C termini (dPEG-24-PA-dPEG24) (31). All three forms were soluble in water, but only PA-dPEG24 retained full inhibition of the classical complement pathway at a level similar to PA in DMSO. Due to the increased aqueous solubility, PA-dPEG24, showed enhanced dose-dependent inhibition of hemolysis compared to PA in the hemolytic assay *in vivo* in rats (31). Additional PA derivatives with decreasing numbers of C-terminal PEG moieties were also created and solubility of the peptide in water was retained when the PEG moiety was reduced from 24 to 2 C-terminal PEG residues, with continued preservation of complement inhibition up to 89% as demonstrated in the ABO incompatibility assay (31). PA-dPEG24 became the lead EPICC compound for further development and is commonly referred to in the literature as Peptide Inhibitor of Complement C1 (PIC1). In order to correctly reference each peptide for the rest of the manuscript, we will use the RLS (ReAlta Life Sciences) numbering system; therefore, PA-dPEG24 will be referred to as RLS-0071 to distinguish it from other EPICC peptides. Below we discuss the ongoing development of EPICC peptides to create advanced forms of RLS-0071 with enhanced solubility and other desirable characteristics. In addition, the unanticipated finding that the EPICC molecules inhibit neutrophil effector functions will be discussed, as well as the current progress in the pre-clinical development of RLS-0071 as a potential therapeutic for human acute inflammatory conditions.

## Mechanisms of Action

### Inhibition of Complement

PA had been previously shown to maintain the functional properties of the parent molecule, the capsid protein of HAstV-1, through binding of C1q to inhibit classical pathway activation as well as binding to the structurally similar proteins mannose binding lectin (MBL) and ficolins (**Figure 1**) (31). HAstV-1 capsid protein inhibits the activation of C1 through binding of C1q and displacement of the C1s-C1r-C1r-C1s tetramer (22, 25). Similarly, PA binds to the CLR of C1q as shown by ELISA and surface plasmon resonance experiments inhibiting activation of the serine protease tetramer (29) but does not displace the tetramer association with C1 likely due to its small size (23, 31). PA was also demonstrated to bind to the CLR of wild-type MBL but not a derivative form of the MBL (K55Q) that is unable to bind its serine proteases MASP-1 and MASP-2 (the functional equivalent of the C1s-C1r-C1r-C1s tetramer) (31, 32). These findings suggest that PA binds to the hinge region of the CLR of both C1q and MBL in a similar manner to functionally prevent their respective serine proteases from activating the classical and lectin complement pathways, respectively.

The aqueous soluble PEGylated version of the PA peptide, RLS-0071, was demonstrated to inhibit the classical complement pathway in human serum as well as rat, mouse, and non-human primate serum in the hemolytic assay in a dose-dependent manner, demonstrating that RLS-0071 retains the cross-species functionality (31). As RLS-0071 was able to inhibit complement activation *in vitro* in rat serum, it was then tested *in vivo*. As with the PA peptide, RLS-0071 administered intravenously was able to inhibit complement activation in an *ex vivo* hemolytic assay 30 seconds after peptide administration and had a functional half-life of four hours *in vivo* in Wistar rats (31).

### Modulation of Neutrophil Effector Functions

In addition to complement inhibition, our laboratory has determined that the EPICC peptides can directly inhibit neutrophil effector functions. These properties are independent of EPICC peptide interaction with complement system. The ability of the EPICC peptides to modulate myeloperoxidase activity (MPO), and inhibit of NETosis, as well as exhibit antioxidant activity are described.

#### Inhibition of MPO Activity

**Figure 2:**
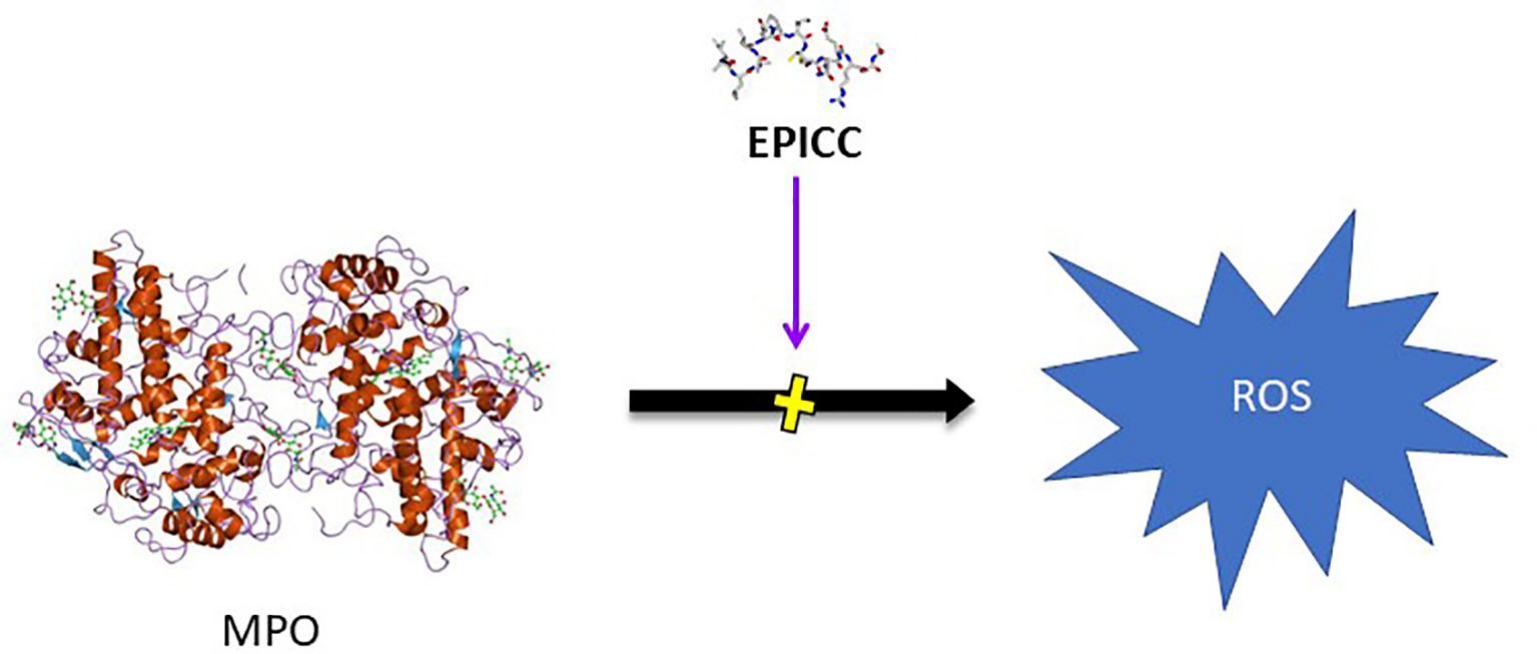
Inhibition of MPO activity by EPICC peptides. MPO released from neutrophils generates reactive oxygen species (ROS) which results in host tissue damage in inflammatory disease conditions. EPICC peptides directly bind MPO and prevent the peroxidase activity of MPO through an antioxidant mechanism that is mediated by cysteine residues in the peptides.

Myeloperoxidase (MPO) is a heme-containing peroxidase enzyme abundantly expressed in the granulocytes of neutrophils. MPO is critical for antimicrobial activity *via* formation of hypochlorous acid (HOCl) from hydrogen peroxide and chloride ion as well as tyrosyl oxide formation from oxidation of tyrosine (33). While these functions are essential for intracellular destruction of bacteria and other pathogens within the neutrophil, upon neutrophil degranulation, MPO is released and causes oxidative damage to host tissue and mediates inflammation through hypochlorous acid and other mechanisms (34, 35). RLS-0071 reversibly and dose-dependently inhibits the peroxidase activity of myeloperoxidase (MPO) that is released from neutrophils as well as purified MPO as assessed in a tetramethylbenzidine (TMB) assay in which TMB serves as the oxidation target (36). The inhibition of MPO by RLS-0071 demonstrates similar potency on a molar basis to the commonly utilized MPO inhibitor, 4-aminobenzoic acid hydrazide (ABAH). RLS-0071 protects the heme ring of MPO from oxidative damage as assessed by spectral analysis and blocks alteration of the iron charge state and release of free iron, which results from the generation of hypochlorous acid, suggesting that the peptide can directly interact with the heme ring within the MPO molecule (**Figure 2**) (36).

Hemoglobin (Hb) peroxidase activity resulting in the production of ROS from free Hb and from iron released from heme degradation can cause severe toxicity to the kidney, liver, central nervous system, and cardiac tissue (37, 38). RLS-0071 inhibited the peroxidase activity of hemoglobin and myoglobin (39) and also that of free Hb released from lysed RBCs. This activity was also tested with purified metHb (oxidized hemoglobin), confirming that the inhibition seen in the RBC lysates was due to inhibition of the Hb. The inhibition of metHb peroxidase activity in a dose-dependent manner by RLS-0071 was similar to ABAH on a molar basis. RLS-0071 and ABAH also inhibited the peroxidase activity of myoglobin in a dose-dependent manner. The inhibition of peroxidase activity of MPO, Hb and myoglobin is attributable to an anti-oxidant mechanism of action mediated by cysteine residues at positions 9 and 10 in RLS-0071 as described below.

#### Antioxidant Activity

**Figure 3:**
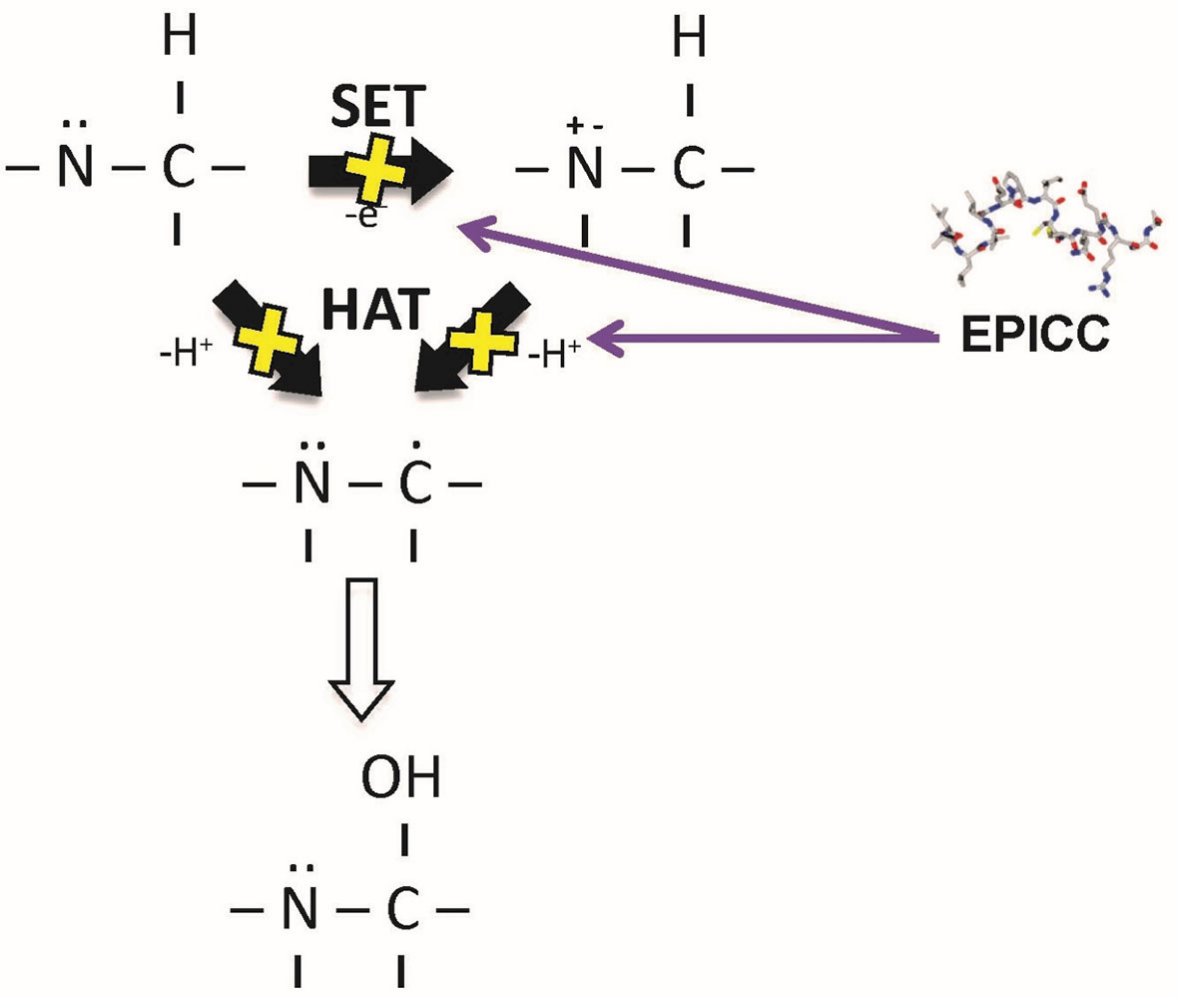
Mechanism of antioxidant activity of the EPICC peptides. EPICC peptides inhibit oxidant formation through the single electron transport (SET) and hydrogen electron transport (HAT) mechanisms. Similar to the cysteine containing tri-amino acid peptide glutathione, EPICC peptide antioxidant activity is mediated by its cysteine residues.

Excessive amounts of reactive oxygen species (ROS) and oxidants can overwhelm natural damage control mechanisms (40) and lead to acute pathogenic events, such as ischemia-reperfusion injury-mediated diseases (41) and hemoglobinemia-mediated acute kidney injury (42). The observed inhibition of peroxidases was consistent with an antioxidant mechanism of action and so the antioxidant potential of RLS-0071 was explored (43). In a total antioxidant activity assay (TAC), RLS-0071 demonstrated dose-dependent antioxidant activity similar to glutathione, a potent cysteine-containing tri-peptide antioxidant that prevents ROS damage by serving as an electron donor for free radicals and heavy metals (44). Oxidation of the vicinal cysteines at positions 9 and 10 in RLS-0071 reduced antioxidant activity significantly in the TAC assay demonstrating that these residues are critical for the antioxidant activity of the peptide. While the activity in the TAC assay showed that RLS-0071 inhibits antioxidant activity through the single electron transfer (SET) mechanism, RLS-0071 was also able to prevent ROS-induced oxidative stress by either the hydroxyl or peroxyl radicals *via* the hydrogen atom transfer process (HAT) as shown in an oxygen radical antioxidant capacity assay (ORAC) and hydroxyl radical antioxidant capacity assay (HORAC) (**Figure 3**).

#### Inhibition of NETosis Activity

**Figure 4:**
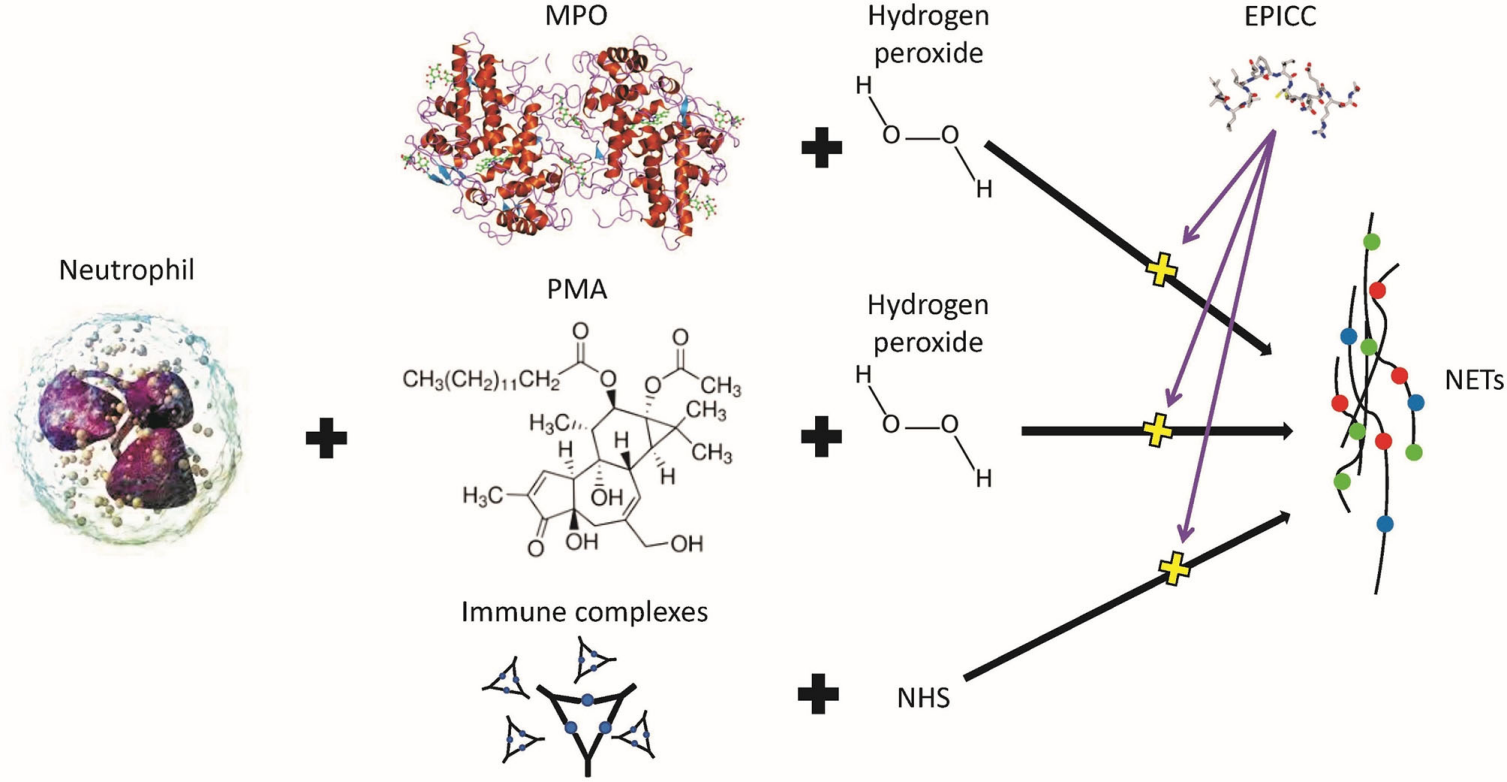
Inhibition of neutrophil extracellular trap (NET) formation is by EPICC peptides. MPO and PMA require hydrogen peroxide to stimulate human neutrophils to produce NETs whereas immune complexes require normal human serum (NHS). EPICC peptides inhibit NET formation by all three of these mechanisms.

Neutrophil extracellular trap (NET) formation is one of the major contributors to the pathogenesis of systemic lupus erythematosus (SLE) and other autoimmune diseases (45–48). NET formation in isolated human neutrophils can be induced with different agents, such as phorbol 12-mystate 13-acetate (PMA) in combination with hydrogen peroxide (H_2_O_2_) as well as immune complexes incubated with normal human serum (NHS) (49). RLS-0071 reduced NET formation by all of these stimuli to baseline levels (50). RLS-0071 inhibition of NET formation is mediated by the sulfhydryl groups of the vicinal cysteine residues of the peptide as oxidation of the cysteine residues with hydrogen peroxide prevents RLS-0071 inhibition of NET formation. MPO has been postulated to be a critical mediator of NET formation through hypochlorous acid (HOCl) production formed by H_2_O_2_ and chloride ions within neutrophils (49). To test this hypothesis, purified MPO combined with H_2_O_2_ induced NET formation in human neutrophils, and this process could be blocked by the addition of RLS-0071 (50) (**Figure 4**). These findings suggest that the inhibition of NET formation is through an MPO-mediated pathway.

**Figure 5:**
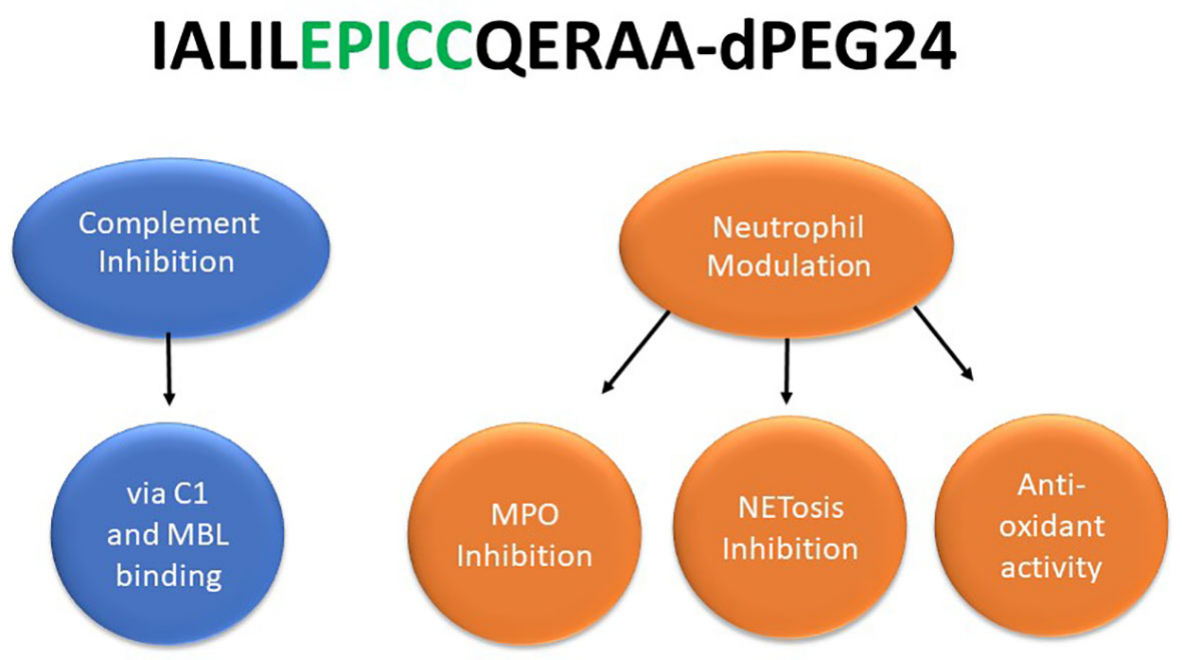
EPICC peptides possess novel dual acting anti-inflammatory activities. The EPICC molecules can block activation of the classical and lectin complement pathways by binding the initiator molecules C1 and MBL, respectively. EPICC peptides also demonstrate inhibition of MPO and NETosis through direct binding of the heme ring of MPO. These peptides also possess antioxidant activity mediate by the vicinal cysteines at positions 9 and 10 of the peptide. The sequence of the 15 residue EPICC molecule, RLS-0071 containing the C-terminal dPEG24 moiety, is indicated.

In summary, these studies demonstrate that EPICC peptides possess dual-acting activities: inhibition of the classical and lectin pathways of the complement system as well as modulation of neutrophil effector functions (NETosis and MPO activity) (**Figure 5**). To our knowledge, molecules with similar dual-acting mechanisms of action have not been previously described in the literature.

## Next Generation EPICC Peptides

The next generation of EPICC peptides developed and studied utilize sarcosine substitutions in the parent PA peptide to increase the aqueous solubility without the need for the PEGylated C-terminal tail (51). Sarcosine residues are frequently used in medicinal chemistry to improve solubility of peptides by reducing the number of intra- and inter-molecular hydrogen bonds and increasing steric constraints caused by altered torsion angles of nearby amino acids (52). Substitution of amino acids A2, L3, I4, L5, I8, C9, and C9,10 with a single sarcosine resulted in peptides that were soluble in water in the absence of PEGylation (51). Compared to the RLS-0071 molecule, this reduced the overall molecular weight of the peptides from 2,771 Da to approximately 1,600 Da. The water-soluble sarcosine peptides were investigated to test their functional activity compared with the parent molecule, RLS-0071. **Table 1** shows the activity of the peptides in the assays tested (51). Most of the peptides retained activity that was similar to RLS-0071. The sarcosine substitution at I8 (RLS-0088) was found to be superior in potency to RLS-0071 for both complement and MPO inhibition. The C9 (RLS-0089) substitution had similar complement inhibition activity to RLS-0071, but the antioxidant capacity was reduced due to the loss of one of the cysteine residues. In contrast, the substitution of both cysteine residues with a single sarcosine residue C9,10 (RLS-0112) was water soluble but lost most of the activity of the RLS-0071 peptide. These findings demonstrate that more potent EPICC molecules may be developed with favorable properties for therapeutic application.

**Table 1:** Summary of EPICC peptide properties.

New Peptide Designations	Historical Designations	Sequence	H_2_0 Solubility	Classical C’ Inhibition	MPO Inhibition	NETosis Inhibition	Total Antioxidant Capacity	Antibacterial Activity
RLS-0071	PA-dPEG24 (PIC1)	H2N-IALILEPICCQERAA-dPEG24	No	++	+++	+++	++++	++
RLS-0082	A2	H2N-I(Sar)LILEPICCQERAA-OH	Yes	++	++++	+++	++	++++
RLS-0083	L3	H2N-IA(Sar)ILEPICCQERAA-OH	Yes	++	+++	++++	+++	++++
RLS-0084	I4	H2N-IAL(Sar)LEPICCQERAA-OH	Yes	++	+++	++	++++	++++
RLS-0085	L5	H2N-IALI(Sar)EPICCQERAA-OH	Yes	+++	+++	++++	++	+++
RLS-0088	I8	H2N-IALILEP(Sar)CCQERAA-OH	Yes	++++	++++	+++	+++	ND
RLS-0089	C9	H2N-IALILEPI(Sar)CQERAA-OH	Yes	++	+++	+++	++	ND
RLS-0112	C9,10	H2N-IALILEPI(Sar)QERAA-OH	Yes	0	0	+	0	ND

Observations in the first (+), second (++) , third (+++), and fourth (++++) quartiles by activity in each assay, with the fourth quartile showing the most activity.

ND, not done.

## Pre-Clinical Development of RLS-0071

The ability of the EPICC peptide RLS-0071 to inhibit both the complement system and neutrophil effector function *in vitro* led us to examine the efficacy of this molecule in a variety of *ex vivo* human studies and in animal models of disease driven by complement and neutrophil-mediated inflammation. In this section, the efficacy of RLS-0071 in blood and tissue-based diseases is discussed.

## RLS-0071 Efficacy in Blood-Based Diseases

### Acute Hemolytic Transfusion Reactions

Transfusion with blood products is lifesaving for many patients but carries the risk of adverse events, some of which can be life-threatening (53). Immune-mediated hemolytic transfusion reactions that occur within 24 hours of the transfusion are known as acute hemolytic transfusion reactions (AHTR) (54). In the case of mismatched RBC transfusion, pre-existing antibodies in the recipient cross react with the donor RBCs resulting in the activation of the classical pathway of complement and hemolysis of the transfused RBCs. Individuals receiving frequent blood transfusions will develop alloantibodies and autoantibodies to RBC antigens over time making agglutinin-based crossmatching performed by blood banking and transfusion services increasingly difficult and thus increasing the risk of AHTRs (54). Agglutination-based cross-matching measures antibody to RBCs but is not able to assess activation of antibody-initiated classical complement pathway-mediated hemolysis (55). To directly measure complement-mediated hemolysis in response to incompatible blood products, our laboratory modified the classical hemolytic complement assay (CH50) to be used with clinical specimens in concert with standard agglutination assays (56). This new assay, the “complement hemolysis using human erythrocytes” (CHUHE) assay, can be measured using serum (CHUHE-S) or plasma (CHUHE-P). The amount of hemolysis that is detected when mixing plasma or sera from type A donors with RBCs from type AB and type B donors was consistent across the hemolytic assays, CHUHE-S, CHUHE-P, and CH50 (56).

The ability to discriminate complement mediated AHTR with the CHUHE assay in a clinical setting was evaluated. A 13-year-old B+ RBC female with sickle cell disease received 3 units of O+ RBCs from two different donors. The patient developed severe anemia consistent with an AHTR (57). The blood from the two donors was found to have elevated anti-B antibodies for both cold and warm agglutination and anti-B IgG. The CHUHE assay determined the blood from one of the two donors generated robust complement-mediated hemolysis thus discriminating different hemolytic activity in the plasma from two RBC donors. To ascertain whether RLS-0071 could inhibit hemolysis induced from the donor plasma mixed with B+ RBCs, the plasma was incubated in the presence of increasing amounts of RLS-0071 peptide and further incubated with B+ RBCs. RLS-0071 demonstrated dose-dependent *ex vivo* inhibition of AHTR hemolysis confirming that the hemolysis detected in the hemolytic complement assay resulted from antibody-initiated classical pathway activation.

Since the hemolysis from the incompatible blood products was found to be inhibited through the addition of RLS-0071 in the CHUHE assay, the potential for RLS-0071 as a prophylactic or rescue treatment was examined in a rat model of AHTR-like intravascular hemolysis after transfusion of incompatible RBCs (58). In this model rats, which have naturally occurring antibodies to human RBCs, receive an incompatible human RBC transfusion and demonstrate rapid antibody-initiated, classical pathway-mediated hemolysis of the transfused RBCs resulting increased levels of free hemoglobin and bilirubin and acute kidney injury. Intravenous infusion of RLS-0071 was able to prevent hemolysis of the incompatible transfused RBCs and decrease the levels of bilirubin formed when administered prior to or 30 seconds after transfusion with mismatched blood products. Additionally, animals receiving RLS-0071 had reduced levels of kidney injury as assessed by histology and gross observations of kidney color and weight (59). These data demonstrate that RLS-0071 may have efficacy as a selective complement inhibitor for the prevention or treatment of AHTRs, which constitute an unmet medical need.

### Delayed Hemolytic Transfusion Reaction

In addition to AHTR, delayed hemolytic transfusion reaction (DHTR) is a rare but life-threatening reaction to blood transfusion (54). While it is thought that many DHTRs are mild and self-limited, severe DHTR reactions may progress rapidly after 1-2 weeks and can be life-threatening (60). The mechanisms underlying DHTR are unclear, but it is commonly accepted that DHTRs are the result of a patient having been previously sensitized to a RBC antigen but possessing undetectable antibodies to this antigen prior to transfusion (61). One to four weeks after transfusion with RBCs that express this antigen, an immune response where the antibody-coated donor cells are thought to be primarily destroyed by extravascular hemolysis in the liver and spleen by Fc-mediated phagocytosis leading to DHTR (54, 62). The role of complement activation in DHTR is unclear. We previously reported that a 14-year-old female patient with sickle cell disease developed a DHTR (63). To ascertain if classical complement pathway activation was contributing to her DHTR, we utilized the (CHUHE) assay and RLS-0071. The patients RBCs and plasma were analyzed in the assay in the presence of increasing amounts of RLS-0071. The peptide dose-dependently decreased hemolysis of the patient’s RBCs suggesting that the classical pathway of complement contributed to her DHTR and suggest that RLS-0071 may have utility to reduce intravascular and extravascular hemolysis in a patient suffering from DHTR.

### Ceftriaxone-Induced Immune Hemolytic Anemia

The CHUHE assay has also been used to evaluate and confirm that complement activation was responsible for other cases of immune-mediated hemolysis such as ceftriaxone-induced immune hemolytic anemia (CIIHA). Ceftriaxone is the second most common agent to cause drug-induced hemolytic anemia and most cases of CIIHA have been documented in children (64). While CIIHA is a rare complication, it can progress to shock, multiorgan dysfunction, and death (65). We previously reported a case of CIIHA in a 10-year-old girl with chronic active Epstein–Barr virus disease and hemophagocytic lymphohistiocytosis (66). After chemotherapy, she was febrile and received ceftriaxone. She rapidly developed respiratory failure and anemia consistent with CIIHA. To confirm her symptoms were consistent with CIIHA, the CHUHE assay was utilized. Exogenous ceftriaxone added to the patient’s serum enhanced lysis of her erythrocytes. RLS-0071 added to the patient’s serum in the presence of ceftriaxone inhibited hemolysis in the CHUHE assay (66).

### Alloimmune Platelet Refractoriness

Alloimmune platelet refractoriness is a condition where repetitive platelet transfusions lead to the development of alloantibodies that bind to transfused platelets such that they are rapidly destroyed and fail to increase a patient’s platelet count resulting in continued thrombocytopenia (67). This increases the risk of bleeding, either through spontaneous events or medical procedures. Alloimmune platelet refractoriness is a significant unmet medical need as the increased risk of bleeding for these patients cannot be corrected by platelet transfusion (68). To understand the role of the classical pathway of complement in platelet refractoriness, *ex vivo* experiments performed by sensitizing human platelets with antibodies from an immune thrombocytopenic purpura patient demonstrated complement opsonization with iC3b and decreased viability in complement-sufficient sera (69). RLS-0071 was able to dose-dependently decrease antibody-initiated complement activation on the platelet surface and increase antibody-sensitized platelet viability in complement-sufficient sera. Increasing human platelet survival in incompatible sera with a classical complement inhibitor represents a novel finding and suggests that pharmacological inhibition of complement activation may have clinical utility in extending transfused platelet viability.

### Autoimmune Hemolytic Anemia in the Setting of Systemic Lupus Erythematosus

Autoimmune hemolytic anemia (AIHA) is caused by the development of anti-erythrocyte antibodies leading to complement activation and is commonly more severe in the setting of systemic lupus erythematosus (SLE) (70–72). To evaluate antibody-mediated complement activation on the surface of erythrocytes, as occurs in AIHA, blood type O RBCs were incubated with sera from subjects with SLE and a history of AIHA which resulted in complement activation as assessed by total C3b, iC3b and C5a generation. RLS-0071 was able to decrease complement activation initiated by these sera samples as measured by reduction of C3-fragment optimization and decreased production of the anaphylatoxin C5a (73).

## RLS-0071 Efficacy in Tissue-Based Diseases

### Cystic Fibrosis

MPO released from neutrophils contributes to inflammatory lung damage in cystic fibrosis (CF) patients. The MPO in cystic fibrosis lung fluid (sputum) generates hypochlorous acid (HOCl) resulting in toxic peroxidase activity and is likely due to neutrophil degranulation, cell death, or neutrophil extracellular trap (NET) formation (74, 75). To ascertain if RLS-0071 could inhibit MPO peroxidase activity from the lung fluid of CF patients, sputum specimens were incubated in the presence or absence of RLS-0071 and the oxidation of TMB assessed as a measure of MPO activity (36). RLS-0071 inhibited MPO activity in CF sputum soluble fractions by an average 3.4-fold decrease in this ex vivo assay. RLS-0071 dose-dependently inhibited peroxidase activity by purified MPO or MPO isolated from neutrophil lysates. Additionally, when incubated with oxidized TMB, RLS-0071 reversed the oxidation state of TMB, as measured by absorbance at 450 nm. This result was consistent with an antioxidant mechanism for PIC1. RLS-0071 inhibition of the high baseline oxidative activity of MPO in the CF sputum soluble fraction, without neutrophils, indicated that this was a direct effect on MPO and complement independent.

### Diabetic Wound Healing

Diabetic wounds can be slow to heal and frequently become chronic (76). This is thought to be due to prolonged inflammation that prevents healing (77, 78). The role of complement-mediated inflammation in diabetic wounds was investigated in a genetic db/db mouse model of diabetes (20). Diabetic mice showed increased concentrations of C5a in the wound fluid and increased C3-fragment staining in the tissue at the edges of the wounds indicating increased complement activation in the wounds of diabetic mice compared to the heterozygous controls. Increased leukocyte infiltration, primarily neutrophils, in the wounds of the diabetic mice was also observed by histopathology. Addition of RLS-0071 decreased C5a in the wound fluid, decreased leukocyte/neutrophil infiltration, and showed a downward trend in C3-fragments in the subcutaneous tissue at the edges of the wound. These findings indicate that there is substantial complement activation in diabetic skin wounds that contribute to persistent inflammation and that complement modulation as demonstrated by RLS-0071 may be a promising therapeutic target.

### Hypoxic Ischemic Encephalopathy

Perinatal hypoxic ischemic encephalopathy (HIE) affects 0.15% of live births in developed countries and up to 1.6% in developing countries (79). Currently, induction of hypothermia by six hours of age is the standard therapeutic intervention (80, 81), which improves survival but only moderately reduces the risk of severe disability (82) and no pharmacological adjunctive therapy currently exists. It has been previously demonstrated that complement-activated inflammation plays an important role in ischemia-reperfusion injury associated with HIE (83–85) as well as MPO and oxidative stress contributing to brain tissue damage (86). To determine if the anti-inflammatory properties of RLS-0071 could reduce brain infarction and improve neurocognitive outcomes, the well-established HIE rat pup model, as described in Shah etal. (80), was utilized where animals received hypoxia and unilateral carotid ligation. Rat pups receiving 2 doses of 10mg/kg RLS-0071 at 1-hour and 4-hours post-hypoxia in combination with hypothermia, showed a 22% increase in neuronal density compared to the hypothermia only group, while both groups receiving RLS-0071 with and without hypothermia exhibited an increase in neuronal density compared to the normothermic control (87). Additionally, cerebral lesion volumes were measured by magnetic resonance imaging (MRI), and lesion volume was significantly decreased in rats given RLS-0071 and hypothermia compared to the hypothermia only controls 24 hours after HIE insult.

Classical complement factor C1q has been implicated as a driver of oxidative stress during HIE injury and C1q deficient mice subject to HIE demonstrate neuroprotection (84). To assess the effects of RLS-0071 on C1q expression in the brain tissue of rats subject to HIE, animals were administered RLS-0071 with and without hypothermia. RLS-0071 decreased C1q levels in the brain compared to the hypothermia only control at one hour post peptide administration. After eight hours, animals given RLS-0071 and hypothermia showed decreased C1q compared to the normothermic group (87).

To determine the effects of RLS-0071 on neurocognitive function, rats subject to the HIE insult with and without RLS-0071 and hypothermic treatment were assessed by Barnes maze and novel object recognition index at six weeks of age. In the Barnes maze test, which measured long-term spatial memory retention (88), rats given RLS-0071 with and without hypothermia spent less time navigating the maze and made fewer errors than the hypothermic control. Additionally, rats were tested with familiar and novel objects, where animals that remember the familiar object will investigate the novel object longer (89). Both groups given RLS-0071 spent significantly more time with the novel object than the familiar one. In summary, these studies show that RLS-0071 targeting C1q along with antioxidant and neutrophil modulating effects can provide additional neuroprotection over hypothermia alone when given as rescue therapy in an animal model of HIE. Mechanistically, these studies suggest that pharmacological intervention inhibiting classical complement pathway activation combined with antioxidant, myeloperoxidase, and NETosis inhibition may hold promise for modifying HIE brain damage.

### Acute Lung Injury

Acute lung injury (ALI) results from underlying inflammation coupled with a secondary insult, such as viral pneumonia or a blood transfusion (90–92) and can lead to acute respiratory distress syndrome (ARDS), which increases morbidity and mortality (93). Improper regulation of neutrophils and the complement system are important modulators in ALI, COPD, and steroid resistant neutrophilic asthma (94, 95). Given the ability of RLS-0071 to inhibit both complement and neutrophil-mediated activation, we developed a two-hit rat model of ALI. In this model, lipopolysaccharide (LPS) is administered IV to adult rats followed by IV transfusion with 30% incompatible RBCs 30 minutes later (43). Four hours after incompatible RBC transfusion, lungs show significant neutrophil-mediated damage along with significant increases in blood levels of complement factor C5a and free DNA which is a biomarker of NETosis (96, 97). Additionally, significant increases in inflammatory cytokines (IL-1a, IL-1b, IL-6, IFN-g, IL-17, IL-18, TNFa) and chemokines (MCP-1, MIP-1a and MIP-2) are observed. Animals receiving a single prophylactic dose of 40mg/kg RLS-0071 either two minutes prior to transfusion or single rescue doses at 0.5 minutes and up to 180 minutes after incompatible RBC transfusion exhibited significant decreases in lung damage, C5a, and free DNA levels and reduction of inflammatory cytokines and chemokines compared to the sham animals (98). A single rescue dose of 10mg/kg was also protective. The potent inhibition of ALI observed in this 2-hit model by RLS-0071 can be attributed to the dual anti-inflammatory activities of the molecule, namely complement inhibition and neutrophil modulation at the earliest stage of immune dysregulation. RLS-0071 has been demonstrated to inhibit classical complement activity within 30 seconds of IV administration in the rat (31) and directly reduces neutrophil activation (NETosis and myeloperoxidase activity) (36, 50, 51). By acting within seconds, RLS-0071 downregulates both the humoral and cellular aspects of the innate immune response at the earliest stage of the inflammatory cascade preventing the cytokine storm and ensuing tissue damage.

## Discussion

As a frontline defense against invading pathogens, complement plays a critical role in neutralizing viruses and destroying infected host cells. The complement system is an ancient arm of the innate immune system with its origins having been traced back 1 billion years (99). Similarly, viruses have evolved various countermeasures to evade the host complement system, such as synthesizing virally-derived complement regulators, pirating of complement regulators to inhibit viral particle activation of the complement system, virally-encoded proteases that cleave host complement proteins, and inhibiting complement protein synthesis (100). In the case of human astrovirus, it has been well established that infection of humans and other animals produces a non-inflammatory gastroenteritis suggestive of direct immune suppression (3). Our initial results demonstrated that a defined region of the capsid protein present on the surface of the virion specifically interacts with the initiator molecules of the classical and lectin pathways of complement, C1q and MBL, respectively, to inhibit complement activation and downstream effector function. While evasion of the complement response has been described for many different virus families (100), to our knowledge astrovirus is the only viral family to directly inhibit complement activation through C1q and MBL.

Further refinement of the complement inhibiting region of the 787 residue HAstV-1 capsid protein identified a 15 amino acid sequence that was then rearranged to yield the PA peptide. To produce a more soluble version of this molecule a 24-mer monodisperse PEG tail was added to yield RLS-0071. RLS-0071 is amphipathic with a hydrophobic N terminus and hydrophilic C terminus. Unlike membrane disrupting peptides that are amphipathic in nature such as antimicrobial peptides (AMP) or cell-penetrating peptide (CPP) and result in cell membrane disruption (101), the EPICC peptides do not disrupt cellular membranes and show no cellular lysis in *in vitro* assays using red blood cells or neutrophils (31, 50). AMPs and CPPs are typically 30-100 amino acids, may have multiple disulphide linkages, commonly have a positive net charge (from +4 to +6), contain a hydrophobic region bestowing an amphipathic nature to this class of peptides and have defined secondary structures that may either helical or beta sheet (101). In contrast, the EPICC peptide RLS-0071 is only 15 residues in length, has a net charge of +1, contains only 2 viscinal cysteine residues and is structurally disordered (31). While having amphipathic character, the length of RLS-0071 compared to AMPs and CPPs suggest it would be sub-optimal for membrane disruption. This along with the lack of positive charges to interact with negatively charged cell membranes and no defined helical or beta sheet secondary structure stabilized by disulphide bonding suggest that RLS-0071 does not disrupt or alter cellular membrane integrity. While not damaging to host cells, the efficacy of RLS-0071 in our pre-clinical tissue-based models (i.e., ALI and HIE) suggest that the hydrophobic amino terminus of RLS-0071 allow for efficient tissue binding without affecting cell membrane integrity. Further, we hypothesize that the hydrophobic amino terminus allows for enhanced interaction with the hydrophobic hinge region of C1q and MBL (29, 31) as well as the hydrophobic core of hemoglobin, myoglobin and MPO (39).

Further studies on RLS-0071 led to the unexpected finding that the molecule also directly inhibit neutrophil effector functions (i.e., myeloperoxidase and NETosis inhibition) and possess antioxidant activity. This dual acting mechanism of action has been preserved in the next generation of EPICC peptides with the sarcosine residue substitutions that show activity similar to or better (i.e., RLS-0088) than RLS-0071 for both complement inhibition and neutrophil modulation. Additionally, these derivatives are soluble without C-terminal PEGylation which decreases the size of the molecule significantly.

The ability of the RLS-0071 to inhibit classical complement activation in multiple species (i.e., mouse, rat, dog and cynomolgus macaques) allowed the study of the peptide not only in human ex vivo studies but also rodent disease models in which complement and neutrophil-mediated inflammation play a role. RLS-0071 shows efficacy in both blood and tissue-based acute inflammatory disease models such as AHTR, HIE, ALI and platelet refractoriness. The efficacy of RLS-0071 in these different pre-clinical models is most likely attributed to the ability of the EPICC molecules to inhibit both the cellular and humoral arms of the innate immune response as opposed to only targeting one inflammatory pathway in concert with rapid inhibition early in the complement cascade and before significant neutrophil activation has occurred. In summary, the EPICC peptides represent a new class of anti-inflammatory compounds that modulate the classical and lectin complement pathways and neutrophil effectors. This unique constellation of anti-inflammatory functionality suggests an opportunity to modulate inflammatory diseases *via* mechanisms that current medications can only address singly or not at all.

## Author Contributions

NK and KC developed the concept, created the tables and figures, and reviewed and edited the article. GP authored sections of the manuscript and proofread the manuscript. All authors contributed to the article and approved the submitted version.

## Conflict of Interest

This work was funded by ReAlta Life Sciences, Inc, an entity that is pursuing therapeutic development of the Peptide Inhibitors of Complement C1 (EPICC molecules) for various clinical applications. https://realtalifesciences.com/. NK, KC, and GP receive salary from ReAlta. Other than the named authors, no other members of ReAlta as the funding entity participated in study design, data collection and analysis, the decision to publish or the preparation of the manuscript. The funder provided support in the form of salaries for authors NK, KC, and GP but did not have any additional role in the study design, data collection and analysis, decision to publish, or preparation of the manuscript. The authors have read the journal’s policy and the authors of this manuscript have the following competing interests: NK, KC, and GP are employees of ReAlta Life Sciences. NK and KC have ownership of ReAlta shares and KC serves as on the Board of Directors of ReAlta Life Sciences. NK and KC are listed as inventors on patents that describe PIC1 molecules. The authors would like to declare the following patents/patent applications associated with this research: 8,241,843 methods for regulating complement cascade proteins using astrovirus capsid protein and derivatives thereof, 15/738,786 synthetic peptide compounds and methods of use, 16/400,486 synthetic peptide compounds and methods of use, 8,906,845 peptide compounds to regulate the complement system, 9,422,337 peptide compounds to regulate the complement system, 10,005,818 derivative peptide compounds and methods of use, 9,914,753 peptide compounds to regulate the complement system, 10,414,799 peptide compounds to regulate the complement system, 16/534,200 peptide compounds to regulate the complement system, 16/242,550 PIC1 inhibition of myeloperoxidase oxidative activity in an animal model.

## Publisher’s Note

All claims expressed in this article are solely those of the authors and do not necessarily represent those of their affiliated organizations, or those of the publisher, the editors and the reviewers. Any product that may be evaluated in this article, or claim that may be made by its manufacturer, is not guaranteed or endorsed by the publisher.
